# Vaginal microbiota diverges in sows with low and high reproductive performance after porcine reproductive and respiratory syndrome vaccination

**DOI:** 10.1038/s41598-020-59955-8

**Published:** 2020-02-20

**Authors:** L. P. Sanglard, S. Schmitz-Esser, K. A. Gray, D. C. L. Linhares, C. J. Yeoman, J. C. M. Dekkers, M. C. Niederwerder, N. V. L. Serão

**Affiliations:** 1 Department of Animal Science, Iowa State University, Ames, 50011 USA; 2 Interdepartmental Microbiology Graduate Program, Iowa State University, Ames, 50011 USA; 3 Smithfield Premium Genetic, Rose Hill, 28458 USA; 4 Department of Veterinary Diagnostic & Production Animal Medicine, Iowa State University, Ames, 50011 USA; 5 Department of Animal & Range Sciences, Montana State University, Bozeman, 59717 USA; 6 Department of Diagnostic Medicine/Pathobiology, Kansas State University, Manhattan, 66506 USA

**Keywords:** Microbiology, Zoology

## Abstract

Previous studies have demonstrated evidence for a relationship between the vaginal microbiome and reproductive performance, suggesting the vaginal microbiota may serve as a tool to predict farrowing outcomes in commercial pigs. In this study, we compared the vaginal microbiome in sows with low and high farrowing performance and used it to classify animals with contrasting reproductive outcomes in commercial sows following immune challenge with porcine respiratory and reproductive syndrome (PRRS) vaccination. Eighteen microbes were differentially abundant (*q*-value < 0.05) between the Low and High farrowing performance groups. Among them, *Campylobacter*, *Bacteroides*, *Porphyromonas*, *Lachnospiraceae unclassified*, *Prevotella*, and *Phascolarctobacterium* were also selected in the discriminant and linear regression analyses, and could be used as potential biomarkers for reproductive outcomes. The correct classification rate in the two groups was 100%. In conclusion, this study demonstrates that vaginal microbiota collected after PRRS vaccination could be potentially used to classify sows into having low or high farrowing performance in commercial herds.

## Introduction

Farrowing performance in commercial sows is a key component of cost-effectiveness in the swine industry. Reproductive technologies (e.g., artificial insemination) have allowed producers to maximize the use of existing resources in the production systems to improve reproductive efficiency^1^. Strategies such as selection have allowed the genetic improvement of sows to exhibit enhanced farrowing performance^2^. However, variation in these traits is little explained by the individual’s genetic, creating challenges for rapid improvements in farrowing performance. Recently, it has been shown that host-associated microbiota plays a role in shaping phenotypes of humans and animals^3^. For example, the vaginal microbiota has been shown to impact preterm birth and neonatal health^4^. In beef cattle, the vaginal microbiota was used to distinguish between heifers that were able to establish pregnancy from those that were not^2^, suggesting its potential to be used to identify animals with favorable reproductive performance. In addition, relationships between the microbiota and immune response have been previously observed^5^. Therefore, microbiota collected after modified live virus (MVL) porcine reproductive and respiratory syndrome (PRRS) vaccination may be an alternative indicator of reproductive performance in commercial sows. In addition, the microbiota is relatively easy to collect and can be profiled with considerable ease using current molecular techniques^6^, making it a potential candidate phenotype to predict reproductive outcomes in commercial sows. Therefore, the objectives of this study were to identify differences in composition and alpha diversity of vaginal microbiota between sows with low and high farrowing performance, and to use the vaginal microbiota to classify animals with contrasting reproductive outcomes following PRRS vaccination.

## Results

### Identification of DAMs and alpha diversity between sows with Low and High farrowing performance

**Figure 1:**
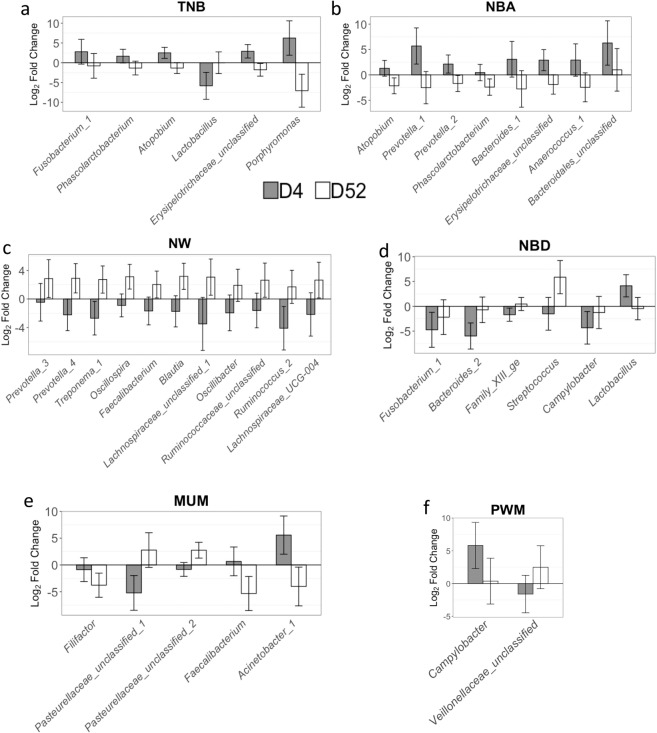
Differentially abundant microbes for the interaction between farrowing performance group and day of microbiome collection. The groups were defined based on the best/worst performance of (**a**) total number born, TNB; (**b**) number born alive, NBA; (**c**) number of weaning, NW; (**d**) number born dead, NBD; (**e**) number of piglets mummified, MUM; and (**f**) pre-weaning mortality, PWM. Positive and negative values represent higher abundance in the groups with high and low farrowing performance, respectively. Bar colors represent collections of days 4 (D4; gray) and 52 (D52; white) after vaccination to porcine respiratory and reproductive syndrome. The errors bars correspond to 95% confidence interval.

**Figure 2:**
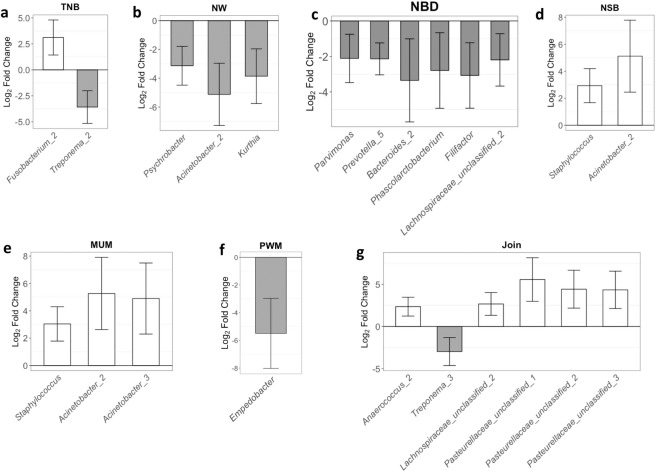
Differentially abundant microbes (DAMs) between groups of animals classified as High or Low farrowing performance. (**a**) Total number born, TNB; (**b**) number weaning, NW; (**c**) number born dead, NBD; (**d**) number of stillborn, NSB; (**e**) number of piglets mummified, MUM; (**f**) pre-weaning mortality; and (**g**) NBA associated with NBD, Join. The errors bars correspond to 95% confidence interval. The colors represent greater (white) or lower (gray) abundance in High performance group compared to the Low performance group.

Differences in microbiota (*q*-value < 0.05) relative abundances were identified for the interaction between farrowing performance group and day of collection (Fig. 1) and for the main effect of farrowing performance group (Fig. 2).

For the interaction, differentially abundant microbes (DAMs) were identified for total number born (TNB), number born alive (NBA), number of piglets weaning (NW), number born dead (NBD), number of piglets mummified (MUM), and pre-weaning mortality (PWM; Fig. 1). For TNB (Fig. 1a), *Fusobacterium_1*, *Phascolactobacterium*, *Atopobium*, *Eryspelotrichaceae*, and *Pophyromonas* had higher abundance in the High farrowing performance group compared to the Low with the log_2_ fold change (log_2_FC) being larger on day 4 (D4) compared to day 52 (D52). In contrast, *Lactobacillus* had lower abundance in the High farrowing group compared to the Low farrowing group on D4 but did not differ on D52. For NBA (Fig. 1b), the identified DAMs *Atopobium*, *Bacteroides_1*, *Phascorlactobacterium*, *Bacteroidales_unclassified*, *Erysipelotrichaceae_unclassified*, *Anaerococcus_1*, *Prevotella_1*, and *Prevotella_2* had higher abundance in the High group compared to the Low group on D4 compared to D52, when the abundance in the Low group compared to the High group increased. For NW (Fig. 1c), *Prevotella_3, Prevotella_4, Treponema_1*, *Oscillospira*, *Faecalibacterium*, *Blautia*, *Lachnospiraceae_unclassified_1*, *Oscillibacter*, *Ruminococcus*, and *Lachnospiraceae_UCG-004* had lower abundance in the High group compared to the Low group on D4. They had higher abundance in the High group on D52. For NBD (Fig. 1d), *Fusobacterium_1*, *Bacteroides_2*, and *Campylobacter* had lower abundance in the High group compared to the Low group on both D4 and D52, but the difference in abundance decreased from D4 to D52. There was lower abundance of *Family XIII* and *Streptococcus* in the High group compared to the Low group on D4 compared to D52, when the abundance in the High group compared to the Low group increased. Finally, *Lactobacillus* was more abundant in the High group compared to the Low group on D4 compared to D52, when the abundance in the High group compared to the Low group decreased. For MUM (Fig. 1e), *Pasteurellaceae_unclassified_1 and Pasteurellaceae_unclassified_2* had greater abundance in the Low compared to the High group on D4 and on D52 the abundance in the High group compared to the Low group increased. *Filifactor* had lower abundance in the High group compared to the Low group on D52 and on D4. *Faecalibacterium* and *Acinetobacter_1* had higher abundance in the High group compared to the Low group on D4 compared to on D52, when the abundance in the Low group compared to the High group increased. For PWM (Fig. 1f), *Campylobacter* had higher abundance on High group on D4 but not on D52 and *Veillonellaceae unclassified* had higher abundance on Low group on D4 and the opposite on D52.

For the main effect of farrowing performance group, DAMs were identified (*q*-value < 0.05) for TNB, NW, NBD, number of stillborn piglets (NSB), MUM, PWM, and Join (representing joint analysis of NBA and NBD), as depicted in Fig. 2. Positive and negative log_2_FCs refer to higher and lower abundance on High compared to the Low farrowing performance groups, respectively. For TNB (Fig. 2a), 2 DAMs were identified: *Fusobacterium_2* (log_2_FC = 3.11) and *Treponema_2* (log_2_FC = −3.57). For NW (Fig. 2b), *Psychrobacter* (log_2_FC = −3.13), *Acinetobacter_2* (log_2_FC = −5.12), and *Kurthia* (log_2_FC = −3.85) showed greater abundance in the Low performance group compared to the High. Six DAMs between sows from High and Low groups were identified when the groups were defined based on NBD (Fig. 2c). For NBD, all DAMs were more abundant in the Low group compared to the High group: *Parvimonas* (log_2_FC = −2.11), *Prevotella_5* (log_2_FC = −2.13), *Bacteroides_2* (log_2_FC = −3.35), *Phascolarctobacterium* (log_2_FC = −2.79), *Filifactor* (log_2_FC = −3.07), and *Lachnospiraceae_unclassified_2* (log_2_FC = −2.19). For NSB (Fig. 2d) and MUM (Fig. 2e), *Staphylococcus* and *Acinetobacter_2* were more abundant in the High group, with log_2_FC of 2.93 and 5.11 for NSB, respectively, and 3.04 and 4.89 (*Acinetobacter_2*) and 5.26 (*Acinetobacter_1*) for MUM, respectively. For PWM (Fig. 2f), *Empedobacter* (log_2_FC = −5.49) was more abundant on the Low group compared to the High. For Join (Fig. 2g), the DAMs were represented by *Anaerococcus_2* (log_2_FC = 2.35), *Treponema_3* (log_2_FC = −2.97), *Lachnospiraceae_unclassified_2* (log_2_FC = 2.66), *Pasteurellaceae_unclassified*_1 (log_2_FC = 5.56), *Pasteurellaceae_unclassified*_2 (log_2_FC = 4.34), and *Pasteurellaceae_unclassified*_3 (log_2_FC = 4.32), being *Treponema_3* the only one more abundant in the Low compared to the High group. No DAMs (*q*-value > 0.05) were identified for NBA. For the effect of day, 180, 250, 106, 8, 60, 41, 96, and 179 DAMs were identified (*q*-value < 0.05) for TNB, NBA, NW, NBD, NSB, MUM, PWM, and Join, respectively (Supplementary Table S1).The operational taxonomic units (OTUs) that were differentially abundant in most analyses performed (5 out of 8) were *Anaerococcus*, with higher abundance on D4 compared to D52 (range log_2_FC = 1.30 to 5.16); *Ruminococcaceae*, with higher abundance on D52 compared to D4 (range log_2_FC = −0.81 to −5.48); *Clostridium sensu stricto*, with higher abundance on D52 compared to D4 (range log_2_FC = −0.79 to −4.56); *Lachnospiraceae* unclassified, with higher abundance on D52 compared to D4 (range log_2_FC = −1.39 to −5.29); and *Prevotellaceae,* with higher abundance on D52 compared to D4 (range log_2_FC = −1.16 to −4.11).

**Figure 3:**
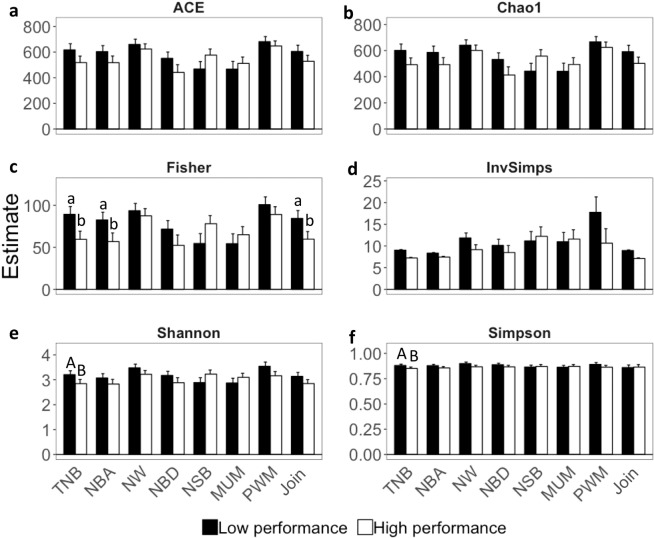
Alpha-diversity measurements for Low and High farrowing performance groups. The alpha-diversity measurements are (**a**) abundance-based coverage estimator (ACE), (**b**) Chao1, (**c**) Fisher, (**d**) Inverse Simpson (InvSimps), (**e**) Shannon, and (**f**) Simpson. Farrowing performance groups were defined based on the best/worst performance of total number born (TNB), number born alive (NBA), number weaned (NW), number born dead (NBD), number of stillborn (NSB), number of piglets mummified (MUM), pre-weaning mortality (PWM), and combination of NBA and NBD (Join). The errors bars correspond to 95% confidence interval. Means lacking common lower- and uppercase letters are statistically different at *P* < 0.05 and <0.10, respectively.

Differences in the microbiota alpha-diversity between farrowing performance groups are presented on Fig. 3. Differences were only observed in Join, NBA, and TNB (*P*-value < 0.05) for Fisher alpha-diversity. There was a higher Fisher alpha-diversity in the Low performance group compared to the High performance. For TNB, there was also a tendency (*P*-value < 0.10) for higher alpha-diversity based on Shannon and Simpson measurements in the Low performance group compared to the High group.

### Classification of gilts into farrowing performance groups based on OTU abundance

The selected OTUs, along with their standardized coefficients, are shown in Supplementary Table S2. The number of selected (*P*-value < 0.05) OTUs ranged from 14 to 16. The OTUs classified as *Lachnospiraceae unclassified* and *Ruminococcaceae* were selected in the analyses based on all traits. *Porphyromonas*, *Campylobacter*, *Fillifactor*, *Prevotella*, *Actinobacillus*, and *Fusobacterium* were selected in at least 3 analyses. All linear discriminant analyses (LDA) had *R*^2^ > 0.99 and *P*-value < 0.001. The leave-one-out cross-validation resulted in a correct classification rate of 100% for all analyses.

The LDA was also performed for each day separately and the selected OTUs along with their standardized coefficients can be seen on Supplementary Table S3. For this, the leave-one-out cross-validation resulted in a correct classification rate of 100% for all analyses, with exception of NBA and MUM on D4, and NW on D52, which had a correct classification rate of 95% (i.e., only one sample was misclassified). In these cases, the accuracies of the classification [i.e., area under the receiver operating characteristic (ROC) curve; AUC] were 0.95, 0.89, and 0.95, respectively, as can be observed on Supplementary Fig. S1.

### Prediction of farrowing performance based on OTU

**Figure 4:**
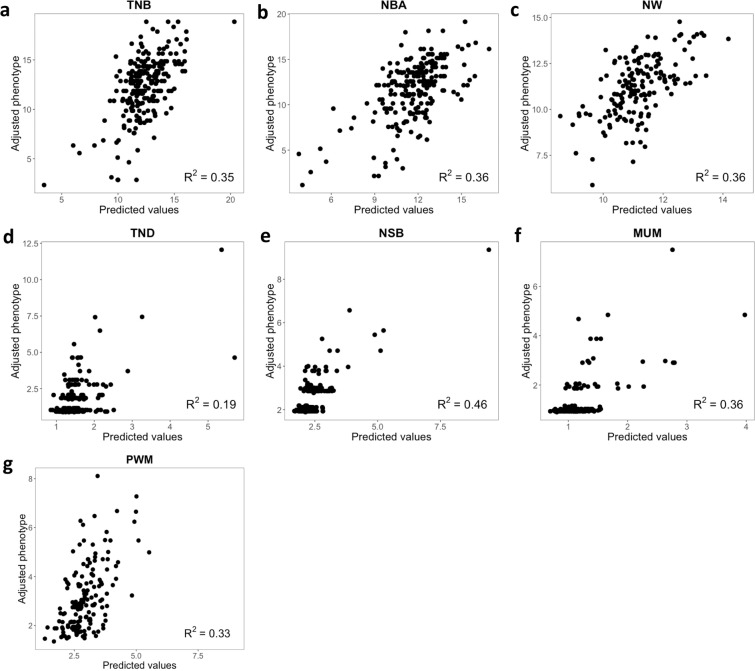
Stepwise linear regression of operational taxonomic units (OTUs) on farrowing performance traits: (**a**) total number born, TNB; (**b**) number born alive, NBA; (**c**) number weaning, NW; (**d**) number born dead, NBD; (**e**) number of stillborn, NSB; (**f**) number of piglets mummified, MUM; and (**g**) pre-weaning mortality, PWM. The x-axis represents the predicted values and the y-axis represents the adjusted phenotype. R-squares correspond to the coefficient of determination (*R*^2^).

The stepwise linear regression (Fig. 4; Supplementary Table S4) reveled that a moderate linear relationship between reproductive traits and vaginal microbiota, with *R*^2^ ranging from 0.19 (NBD) to 0.46 (NSB). The number of OTUs selected (*P*-value < 0.15) ranged from 4 (NBD) to 16 (NSB) across both days and the most frequently included OTUs were *Lachnospiraceae, Ruminococcaceae, Ruminiclostridium, Subdoligranulum*, and *Alloprevotella*, which were selected for 6, 5, 2, 3, and 2 analyses, respectively. These analyses were also performed for each day separately and can be seen on Supplementary Table S5 and Supplementary Fig. S2.

## Discussion

Previous studies have shown that antibody response to PRRS virus following PRRS infection is associated with reproductive outcomes during natural PRRS infection^7,8^. In addition, a relationship between the microbiota and immune response has been observed^5^. We, therefore, hypothesized that microbiota collected after MLV PRRS vaccination may be an alternative indicator of reproductive performance in commercial sows. In addition, the vaginal microbiota has been shown to be associated to reproductive outcomes in humans and cattle^4^ and has the advantage of being relatively easy to collect and possible to be collected at early ages. Therefore, in this work, we assessed and corroborated the possibility of using vaginal microbiota to identify animals with contrasting reproductive performance in commercial sows.

The vaginal microbiota was collected at 4 and 52 days following the MLV PRRS vaccination. The development of immune response to PRRS occurs late, with production of neutralizing antibodies peaking at 9 to 11 weeks (Lopez & Osorio)^9^. However, initial response can be observed as early as 2 days post infection with the induction of interleukin-10 (IL-10) associated with the generation regulatory T cells. In addition, PRRS outbreaks can be identified within a few days. Thus, by investigating the microbiota on days 4 and 52 after vaccination, we focused on collection of data soon after a PRRS MLV challenge has occurred (day 4), as well during peak antibody response (day 52)^10^. In this study, we had no intention to assess the effect of vaccination on the microbiota, which would have required evaluation of a control group on non-vaccinated gilts; however, we recognize that, given the relationship between the microbiota and immune response^11^, the infection with PRRS virus through vaccination during period of collection may be playing a role in the modulation of the vaginal microbiota. It should be noted that the interactions identified between days of collection and farrowing performance group on OTU abundance may be the result of modulation of the immune response on the microbiota, together with the effect of time and age.

The microbiota has been associated with several phenotypic outcomes (i.e., diseases, stress, reproduction); however, the mechanisms involved in the interaction between the host and the microbiota is still unclear. One possible mechanism that relates the microbiota to reproduction appears to involve hormones^12^. Indeed, the gut microbiota can produce and secrete hormones, while hormones can stimulate or inhibit the development of specific microbes, characterizing a bidirectional relationship^12^. Previous studies of the gut microbiota in mice have found correlations of some of the microbes identified in this study, such as *Lachnospiraceae, Clostridium, Prevotella*, and *Ruminococcaceae*, with circulating hormones (i.e., leptin and urinary estrogen;^13^). Also, *Oscillibacter* has been associated with higher levels of glucocorticoid in the gut microbiota of gorillas^14^. Thus, exploring the host microbiota and relating it to phenotypes of interest could help the swine industry improve animal reproductive performance.

A total of 50 unique DAMs were identified across all analyses. Of these, *Phascolarctobacterium, Filifactor, Atopobium, Campylobacter, Staphylococcus, Treponema, Erysipelotrichaceae unclassified, Acinetobacter, and Faecalibacterium* were identified in multiple analyses. *Phascolarctobacterium*, which was identified in the analyses based on TNB, NBA, and NBD, was more abundant in the Low performance group. Similarly, relative abundance of *Phascolarctobacterium* was negatively correlated with the litter weight of piglets at day 21 of lactation^15^ and higher abundance of *Phascolarctobacterium* was associated with gestational diabetes mellitus in pregnant women (Cortez *et al*., 2018). Uterine *Filifactor*, which had higher abundant in the Low group compared to the High group, as showed in the analyses based on NBD and MUM, has been demonstrated to be a predictor of metritis in dairy cows^16^. In addition, there are evidences that *Filifactor* and *Campylobacter* are important oral pathogens and are associated with infection-related preterm birth in humans^17^. *Staphylococcus* was more abundant in the High group in the analyses based on NSB and MUM, contrasting with some finding in the literature which associated high *Staphylococcus* abundance with lower pregnancy rate in humans^18^. This bacteria genus was identified as a DAM in the analyses of NSB and MUM. Many species of *Treponema* have been reported to be a pathogenic bacteria^19^. This bacterium was more abundant in the Low group as in the analyses of TNB and Join. To the best of our knowledge, there is no information associated with reproductive performance on *Erysipelotrichaceae* unclassified, *Acinetobacter*, and *Faecalibacterium*. Other bacteria, such as *Fusobacterium*, *Bacteroides*, and *Lachnospiraceae*. *Fusobacterium*, which was identified to be more abundant in the High group when the groups were defined based on TNB, is a dysbiosis-associated pathogen^20^. *Bacteroides* and *Lachnospiraceae* were more abundant in the Low group compared to the High when the groups were defined based on NBD. *Bacteroides* has been associated with bacterial vaginosis infertility in humans and reproductive disorders in cattle^2^. *Lachnospiraceae* has been shown to be associated with prediction of pregnancy status^2^ and, similar to *Phascolarctobacterium*, its abundance was negatively correlated with the litter weight of piglets at day 21 of lactation^15^. In general, there was a higher abundance of noxious bacteria in the Low performance group compared to the High. Interestingly, among these bacteria genera, the abundance of *Bacteroides* and *Phascolarctobacterium* in the vaginal microbiome of commercial gilts has been shown to be moderately heritable using the same data presented in this study^21^, suggesting the possibility of performing genetic selection over specific bacteria in the microbiota. For the effect of time, the great majority of the microbes differentially expressed between days had higher relative abundance on D52 compared to D4, including the main microbes that were identified in several analysis: *Ruminococcaceae*, *Clostridium sensu stricto*, and *Lachnospiraceae*. Factors such as age and development of immune defense against the virus after vaccination may be associated with these changes; however, they are completely confounded limiting our conclusion on these findings.

Greater microbiota diversity was observed in the Low group compared to the High group for some of the traits used to define groups. These differences were identified for Fisher, Shannon and Simpson diversities, which account for the total number of species, the proportions of those species, and community evenness. Interestingly, Wang *et al*.^15^ reported low diversity in the group of sows with low litter performance based on the litter weigh at 21 days of lactation. Therefore, it is possible that a higher abundance of noxious bacteria in the vaginal microbiota of Low performance sows generated the increase in alpha-diversity in this group. On the other hand, in humans, bacterial vaginosis, which decreases fertility, has been associated with greater alpha-diversities^22^.

The LDA revealed that the vaginal microbiota was able to distinguish between Low and High groups of farrowing performance. When using microbiome information from both days, all LDA had correct classification rates of 100%, indicating that the use of OTUs to classify animals with contrasting farrowing performance is very promising. For the analyses performed by day, the worst accuracy of classification (AUC = 0.89) was obtained when the groups were based on MUM (using D4 OTU data). This may be due to the fact that the average farrowing performance between the two groups was not very different for this trait (1.1 vs. 0 piglets for the Low and High-performance groups, respectively). In addition to MUM, NBA (using D4 OTU data) and NW (using D52 OTU data), had one misclassified sample (out of 20). In two of these cases (NBA and NW), a sample from the High performance group was incorrectly classified into the Low performance group sample, suggesting that the chance of a gilt with Low performance being classified as High performance is minimal. The bacteria with higher influence on classification into groups in the LDA were very similar to those identified as DAMs in the univariate analyses: *Campylobacter*, *Bacteroides*, *Porphyromonas, Lachnospiraceae unclassified, Anaerococcus, Filifactor*, and *Prevotella*. This suggests that these bacteria could be used as potential biomarkers between the Low and High performance groups.

The use of vaginal microbiota to classify gilts into farrowing performance groups would enhance the process of culling, allowing the identification of low reproductive producers at an early age prior to breeding. The classification based on the microbiota data from D4 had slightly worse performance than from D52; however, this difference was not large enough to discard the possibility of obtaining the microbiota earlier measures of the vaginal microbiome for diagnostic purposes. Therefore, collection on D4 seems to be promising to identify gilts with better future farrowing performance. In addition, vaginal swabs collected after PRRS virus vaccination shown to be a good tool to obtain the microbiota since it is minimally invasive and can be performed at early ages.

The LDA was effective in identifying additional influential OTUs that were not identified in the univariate analyses (i.e., identification of DAMs). The LDA is a multivariate approach, which allows the relationship within the microbiota to be accounted for, enhancing the understanding behind the association between the microbiota and phenotypes of interest.

**Figure 5:**
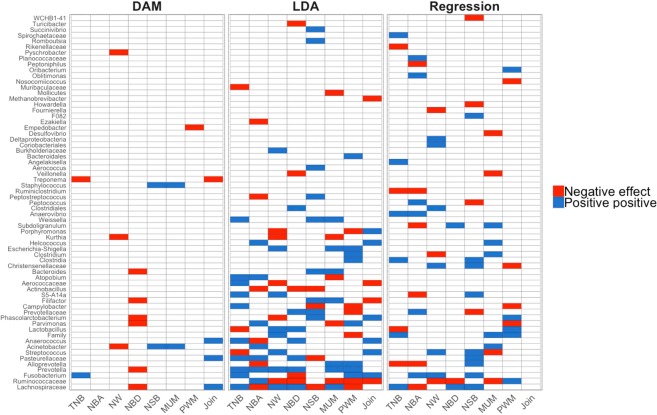
Overall view of the bacteria genera identified for each analysis: differentially abundant microbes (DAM), linear discriminant analyses (LDA), and stepwise regression (Regression). Blue and red represent the positive and negative effects, respectively, of a given bacteria on the farrowing performance: total number born, TNB; number born alive, NBA; number weaned, NW; number of stillborn, NSB; number born mummified, MUM; number born dead, NBD; pre-weaning mortality, PWM; combination of NBA and NBD (Join).

The stepwise regression analyses showed that there is a moderate relationship between vaginal microbiota and farrowing performance, with the vaginal microbiota explaining up to 46% (i.e., NSB) of the variation (i.e., *R*^2^) in subsequent farrowing performance. *Lachnospiraceae* was identified in most of the analyses (6 in total), corroborating the relationship of this bacteria general with farrowing performance in pigs^15^ (Fig. 5). In addition, *Ruminococcaceae*, *Subdoligranulum*, and *Alloprevotella* were selected in most (~four analyses) of the stepwise regression analyses (Fig. 5). These results were not as promising as those from the LDA. While sows with extreme performance were used for the LDA, all animals were used to perform regression analyses of OTUs on farrowing performance. Hence, in these analyses, we evaluated the impact of OTUs across all values of performance. Therefore, based on these results, although there is potential in identifying contrasting performance with OTU data, the use of these data needs to be further evaluated before they can be used to predict overall farrowing performance.

The vaginal microbiota of commercial replacement gilts was associated with subsequent farrowing performance. In this study we identified major differentially abundant microbes in vaginal samples obtained at the commercial level between gilts that were subsequently classified as having Low and High farrowing performances. Gilts with lower subsequent reproductive performance had higher microbiota diversity, suggesting a state of dysbiosis in low-performing animals. High accuracy (100%) can be obtained by using vaginal microbiome data to classify sows into Low and High farrowing performance groups. In contrast, the vaginal microbiota had limited impact in explaining variation in reproductive performance when the whole data set was used, with *R*^2^ up to 0.46 for number of stillborn. Particularly, few genera, such as *Campylobacter*, *Bacteroides*, *Porphyromonas*, *Lachnospiraceae unclassified*, *Prevotella*, and *Phascolarctobacterium* were identified as potential biomarkers of farrowing performance in pigs. In conclusion, this study demonstrates that there is potential in using vaginal microbiota data collected after MVL PRRS vaccination to classify sows with Low and High farrowing performance. Future work should focus on validating bacteria associated with farrowing performance identified in this study, evaluating classification of animals with contrasting performance across multiple parities, as well evaluating the use of vaginal microbiota data collected at different time points and without vaccination.

## Methods

All methods described in this study were approved by the Institutional Animal Care and Use Committee at Iowa State University (IACUC# 6-17-8551-S), following the guidelines and regulations according to the Animal Welfare Act (AWA).

### Animals, sample collection, and reproductive performance traits

Ninety-six F1 (Landrace x Large White) replacement gilts from a commercial farm in North Carolina, USA, were vaccinated (133 ± 11 days old) intramuscularly with a commercial MLV PRRS vaccine (Ingelvac PRRS MLV, Boehringer Ingelheim Animal Health), following the manufacturer’s guidelines. Prior to sample collection, the vulva was cleaned to minimize contamination from skin bacteria into the vagina using water and 70% ethanol. Vaginal swabs were then collected from all gilts using ESwabs (COPAN Diagnostics Inc., Murrieta, CA) at D4 and D52 after vaccination. These 96 animals were selected from a larger pool of 302 sows (described in Sanglard *et al*.^21^) that had first-farrowing performance from January to June 2018 (~150 days post second microbiome collection). In order to work with a more homogeneous dataset (with regards to the time of farrowing), we identified a narrower period of time (March 2018) that included a larger subset of these 302 animals. Subsequent analyses were done using this subset of 96 animals. These animals had data recorded for NBA, NW, MUM, NSB, and PWM. Number born dead was calculated as the sum of MUM and NSB, and TNB was calculated as the sum of NBA and NBD. Summary statistics of the data are presented in Table 1.

**Table 1:** Descriptive statistics of the farrowing performance data.

Traits^a^	N^b^	Mean	SD	Min	Max	Mean (SD) per group^c^	
						Low (n = 10)	High (n = 10)
TNB	96	12.4	3.1	2	19	6.4 (2.5)	17.7 (1.3)
NBA	96	11.6	3.2	1	19	4.4 (2.3)	16.5 (1.4)
NW	93	11.5	1.7	5	15	9.7 (0.7)	13.3 (0.8)
NBD	96	0.8	1.4	0	12	5.1 (2.7)	0.0 (0.0)
NSB	96	0.5	0.9	0	8	3.4 (1.9)	0.0 (0.0)
MUM	96	0.3	0.8	0	7	1.1 (1.6)	0.0 (0.0)
PWM	69	2.7	1.8	1	10	5.1 (2.7)	1.1 (0.3)

^a^TNB, total number born; NBA, number born alive; NW, number weaned; NBD, number born dead; NSB, number of stillborn; MUM, number born mummified; PWM, pre-weaning mortality;

^b^N: number of records.

^c^Mean and standard deviation (SD) from Low and High farrowing performance groups.

### Vaginal microbiota data

The vaginal microbiota data has been previously described in Sanglard *et al*.^21^. DNA from the vaginal swabs was extracted to profile the vaginal microbiota by 16S rRNA gene sequencing. Bacterial DNA was extracted using the Qiagen DNeasy PowerSoil (QIAGEN Inc., Valencia, CA), extraction kit. Briefly, PCR amplicon libraries targeting variable region 4 (V4) of the 16S rRNA gene were produced using a barcoded primer set adapted for Illumina HiSeq. 2000 and MiSeq.^23^. DNA sequence data were generated using Illumina MiSeq paired-end sequencing at the Environmental Sample Preparation and Sequencing Facility (ESPSF) at Argonne National Laboratory (Lemont, IL). Specifically, 515F and 806R primers that included the sequencer adaptor sequences used in the Illumina flow cell were used to PCR amplify the V4 region of the 16S rRNA gene^23^. The 515F amplification primer also contained a twelve base barcode sequence that supported pooling and subsequent demultiplexing of up to 2,167 samples in each lane were included^23,24^. Each 25 µL PCR reaction contained 9.5 µL of MO BIO PCR Water (Certified DNA-Free), 12.5 µL of QuantaBio’s AccuStart II PCR ToughMix (2x concentration, 1x final), 1 µL Golay barcode tagged forward primer (5 µM concentration, 200 pM final), 1 µL reverse primer (5 µM concentration, 200 pM final), and 1 µL of template DNA. The conditions for PCR were as follows: 94 °C for 3 minutes to denature the DNA, with 35 cycles at 94 °C for 45 s, 50 °C for 60 s, and 72 °C for 90 s; with a final extension of 10 min at 72 °C to ensure complete amplification. Amplicons were then quantified using PicoGreen (Invitrogen) and a plate reader (Infinite 200 PRO, Tecan). Once quantified, volumes of each of the products were pooled into a single tube so that each amplicon was represented in equimolar amounts. This pool was then cleaned up using AMPure XP Beads (Beckman Coulter), and then quantified using a fluorometer (Qubit, Invitrogen). After quantification, the molarity of the pool was determined and diluted down to 2 nM, denatured, and then diluted to a final concentration of 6.75 pM with a 10% PhiX spike for sequencing on the Illumina MiSeq. Amplicons were sequenced on a 151 bp MiSeq run using customized sequencing primers and procedures^23^. Mothur was used for sequencing analysis^25^, which followed mothur’s MiSeq standard operating protocol. Barcode sequences, primer and low-quality sequences were trimmed using a minimum average quality score of 35, with a sliding window size of 50 bp. Chimeric sequences were removed using Chimera Uchime. For alignment and for taxonomic classification, the SILVA SSU NR reference database v132^26^ provided by the mothur website was used. The sequences were clustered into OTUs with a cutoff of 99% 16S rRNA gene similarity. The OTUs were numbered in order of abundance (i.e., OTU1 corresponds to the most abundant OTU). More than 2,000 OTUs were obtained, but after removing OTUs that were absent in more that 10% of the samples, 1,386 OTUs were used for microbial analyses. For analyses purposes, the relative abundance of the OTUs was calculated by dividing the counts of each OTU by the total number of counts for a given sample. Alpha-diversity was obtained for each day separately, and measured as: Chao1, inverse Simpson, Simpson, Shannon, Fisher, and abundance-based coverage estimator. Analyses of microbiota diversity were performed in R^27^ using the *microbiome* package^28^.

### Statistical analyses

#### Identification of animals with contrasting reproductive performance

In order to assess the difference in microbial composition and diversity between sows with contrasting farrowing performance, the sows were split into two farrowing performance groups (Low and High). The phenotypes NW and PWM were pre-adjusted for the number of piglets fostered at birth by adding this in the model. Then, sows were identified as Low (bottom 10) or High (top 10) performers after being ranked based on each of these traits separately: NBA, NBD, TNB, NW, MUM, NSB, PWM, and a combination of high farrowing performance for NBA and NBD (Join). Join was created by double-sorting the sows by the NBA and, then, by the NBD in opposite directions to obtain groups with high and low farrowing outcomes. Since microbiota data were collected at D4 and D52, a total of 40 samples (20 animals with two samples representing each time point) were used in these analyses (combination between farrowing performance group and collection day). The average farrowing performance of each group is given in Table 1.

#### Identification of differentially abundant microbes (DAM), and assessment of microbial alpha-diversity between sows with Low and High farrowing performance

For the identification of DAMs, the following repeated measurements negative binomial mixed model was used:

1 $${y}_{ijk}=\mu +grou{p}_{i}+da{y}_{j}+{(group\ast day)}_{ij}+{\beta }_{1}\,ag{e}_{k}+anima{l}_{k}+log({L}_{ijk})$$

where *y*_*ijk*_ is the raw count for the OTU analyzed; *µ* is the overall mean; *group*_*i*_ is the fixed effect of the *i*^th^ farrowing performance group (Low or High); *day*_*j*_ is the fixed effect of the *j*^th^ day of collection (D4 or D52); *β*_1_ is the partial regression coefficient for the covariate age at collection for the *k*^th^ animal (*age*_*k*_); *animal*_*k*_ is the random effect of the *k*^th^ animal, assuming $animal\sim N(0,\,{\boldsymbol{I}}{\sigma }_{animal}^{2})$, where ***I*** is the identity matrix; and *log*(*L*_*ijk*_) is the TMM-normalized library size, used as an offset. The TMM normalization^29^ factors used to normalize library size were obtained based on all the OTUs in the dataset (~2,000) and all animals that had vaginal microbiota data collected (~300; data not shown). A false-discovery rate correction was applied for multiple testing correction^30^, and DAMs for farrowing performance groups were identified when *q*-value < 0.05. Results are being presented as log_2_ fold change (log_2_FC) of the High group compared to the Low (i.e., positive numbers correspond to greater abundance in the High group).

For alpha-diversity, a linear mixed model including the same effects in Eq. 1 was used, with the exception that the offset was removed from the model. The Shapiro-Wilk test for normality was applied and, when the normality assumption of the residuals was not met (*P*-value < 0.05), the response data was log-transformed^31^. Analyses were performed using the GLIMMIX procedure of SAS 9.4 (Statistical Analysis System; Cary, NC, USA), and the calculation of *q*-values were done with the *p.adjust* function from the *stats* package in R.

#### Classification of farrowing performance based on OTU relative abundance

Linear discriminant analyses were performed using the OTU data to classify animals into the two farrowing performance groups (i.e., Low and High groups). For the analyses, microbiome information from D4 and D52 were used to classify into farrowing groups. In addition, analyses were done separately for D4 and D52 in order to optimize the classification on animals.

Prior to LDA, the relative abundance of each OTU was calculated as the proportion of a given OTU divided by the library size for each animal and, then, pre-adjusted for the fixed effect of the covariate age. Next, a stepwise linear discriminant analysis was performed over the adjusted phenotype in order to identify significant (*P*-value < 0.05) OTUs to be included in the discriminant model using stepwise selection. After selection of OTUs, a leave-one-out cross-validation was used to assess the predictive ability of the OTUs to correctly classify samples into the correct groups (Low or High). A ROC curve was used to assess the accuracy (i.e., AUC) of the binary classifier diagnostic. The ROC curve was created by plotting the true positive rate (sensitivity) against the false positive rate (1 – specificity) at various threshold settings. LDA were performed using the STEPDISC and DISCRIM procedures of SAS 9.4. The AUC was calculated with *pROC* package^32^ from R.

#### Prediction of farrowing performance based on OTU

A stepwise linear regression of OTUs on reproductive traits was performed to assess the overall relationship between OTU abundance and farrowing performance. Prior to analyses, the data were pre-adjusted to remove systematic effects. In this step, the reproductive data were adjusted for the fixed effects of farrowing contemporary group (combination of month/year of farrow and farm) and the relative abundance OTU data were adjusted for the covariate of age at microbiota collection. Afterwards, the pre-adjusted reproductive data were used as response variable, one at a time, and the pre-adjusted data of all OTUs were used as explanatory variables. The OTUs were selected to enter and remain in the model in a stepwise approach, using a threshold of *P*-value < 0.15. Analyses were performed using the GLMSELECT procedure of SAS 9.4.

## Supplementary information

Supplementary Figures

Supplementary Tables
