# Supplementary material for: Vaginal microbiota diverges in sows with low and high reproductive performance after porcine reproductive and respiratory syndrome vaccination
Source: Sci Rep. 2020 Feb 20;10:3046. doi: 10.1038/s41598-020-59955-8 (PMC7033195; doi:10.1038/s41598-020-59955-8)
Supplement: Supplementary file 1 — Supplementary Figures [file 41598_2020_59955_MOESM1_ESM.pdf]

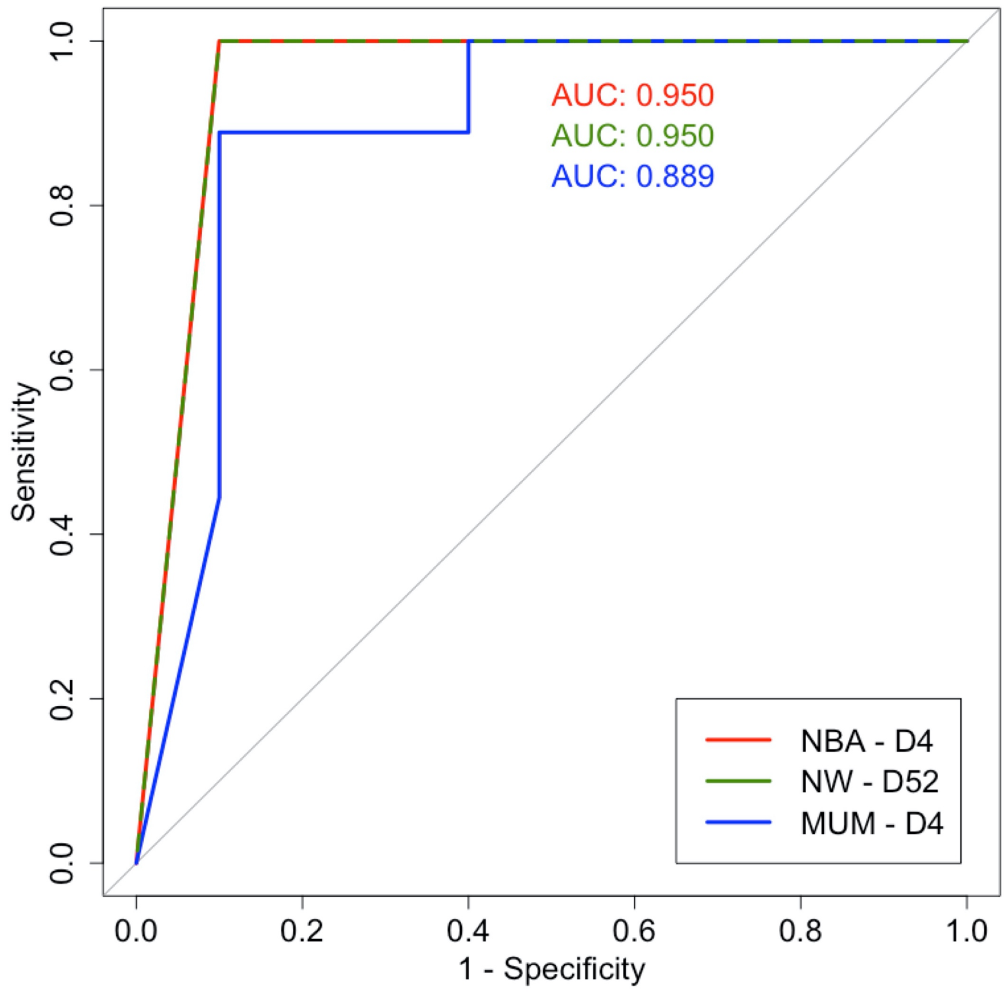

Supplementary Figure S1: Receiver operating characteristic (ROC) curve for the classification in Low and High farrowing performance groups obtained with the leave-one-out cross validation (LOOCV) discriminant analysis for day 4 (D4) and 52 (D52) of collection after vaccination to porcine respiratory and reproductive syndrome. The y-axis (sensitivity) correspond to the true positive rate and the x-axis, false positive rate (1 – specificity) at various threshold settings. The area under de curve (AUC) represents the accuracy of the binary classification. The colors represent results for number born alive (NBA; red), number of piglets mummified (MUM; blue), and number weaning (NW; green). For all other traits and collection days, results are not shown since the classification rate of the LOOCV was of 100%; hence, AUC=1.

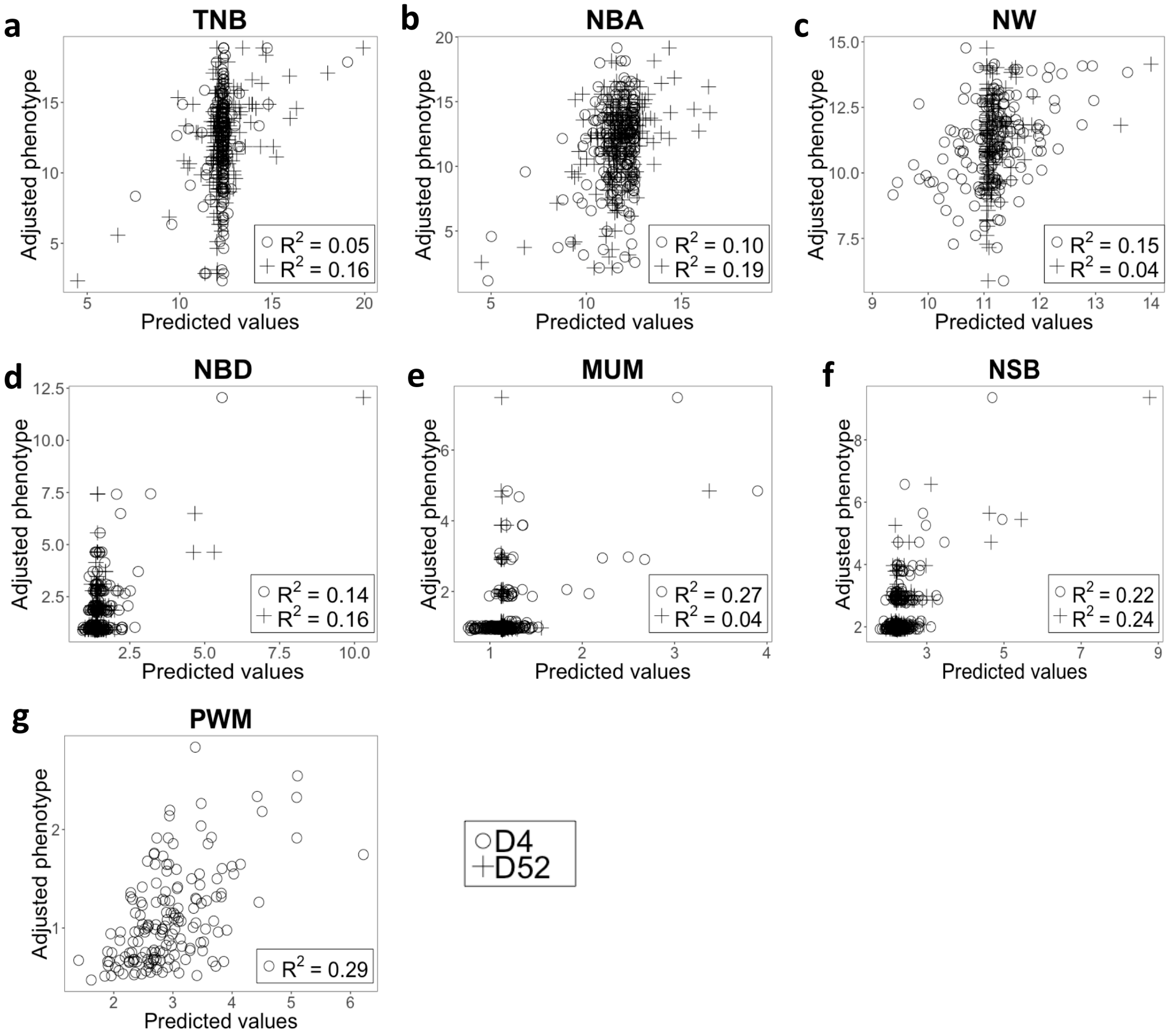

Supplementary Figure S2: Stepwise linear regression of operational taxonomic units (OTUs) on farrowing performance traits: (a) total number born, TNB; (b) number born alive, NBA; (c) number weaning, NW; (d) number born dead, NBD; (e) number of piglets mummified, MUM; (f) number of stillborn, NSB; and (g) pre-weaning mortality, PWM. The x-axis represents the predicted values and the y-axis represents the adjusted phenotype. Circles and crosses represent OTUs collected 4 (D4) and 52 (D52) days after vaccination to porcine respiratory and reproductive syndrome, respectively. The coefficient of determination (R2) was calculated for each day separately (D4 or D52).
